# The effect of magnesium alone or its combination with other supplements on the markers of inflammation, OS and metabolism in women with polycystic ovarian syndrome (PCOS): A systematic review

**DOI:** 10.3389/fendo.2022.974042

**Published:** 2022-08-05

**Authors:** Ruiyun Li, Zhiyuan Li, Yi Huang, Kaiyan Hu, Bin Ma, Yuan Yang

**Affiliations:** ^1^ The First Clinical Medical College, Lanzhou University, Lanzhou, China; ^2^ Gansu Provincial Maternal and Child Health Hospital, Lanzhou, China; ^3^ Evidence Based Medicine Center, School of Basic Medical Sciences, Lanzhou University, Lanzhou, China; ^4^ The reproductive Medicine Center, The 1st Hospital of Lanzhou University, Lanzhou, China

**Keywords:** magnesium, polycystic ovarian syndrome, inflammation, oxidative stress, insulin resistance, lipid profile

## Abstract

The objective of this systematic review is to synthesize the available evidence on the effectiveness of magnesium supplements on the markers of inflammation, oxidative stress (OS), and metabolism in PCOS patients and to provide a basis for its clinical treatment. Electronic databases (PubMed, Cochrane Library databases, Embase, Web of science, CMB, CNKI, VIP, Wan Fang and ClinicalTrials.gov) were searched from their inception until January 2022. Randomized controlled trials (RCTs) for PCOS undergoing therapy with magnesium supplementation alone or in combination with other agents. The primary outcomes were the markers of blood glucose and OS.363 patients from nine RCTs were included in the current systematic review. Four of the nine studies reported the effects of magnesium supplementation alone on OS or metabolic markers in women with PCOS. Whilemagnesium supplementation alone did not show any significant improvement in the markers of inflammation, OS or metabolism in PCOS, seven of the nine articles reported the effect of magnesium co-supplementation on OS or metabolic markers in PCOS patients. Magnesium combined with vitamin E or zinc-calcium-vitamin D significantly improved glucose and lipid metabolism in PCOS patients. Magnesium intake alone did not lead to a significant improvement in the markers of OS, blood glucose, or serum lipids in PCOS. However, magnesium combined with other supplements (vitamin E, zinc, zinc-calcium-vitamin D) significantly improved serum hs-CRP, insulin, HOMA-IR, TG, TC levels, and the improvement in OS markers was inconclusive. The effect of magnesium and melatonin supplementation on the markers of metabolism needs to be further verified.

**System Review Registration:**

PROSPERO https://www.crd.york.ac.uk/PROSPERO/#myprospero, CRD42022303410.

## Introduction

PCOS is one of the most common endocrine diseases in women of reproductive age (1). The main clinical risk associated with PCOS is infertility due to ovulation disorders. The long-term complications of PCOS mainly include diabetes, cardiovascular disease and metabolic syndrome (1, 2). A previously published meta-analysis showed that the pooled prevalence estimate was 5%-15% when PCOS was diagnosed with the Rotterdam criteria (2, 3). Large number of studies have suggested that OS, insulin resistance and dyslipidemia were closely associated with PCOS (4, 5). A previous systematic review had assessed the markers of circulating OS markers in patients with PCOS and control subjects, and reported an increase in the levels of malondialdehyde (MDA) (1.9, CI 95% 1.2 to 2.6) and a reduction in the levels of glutathione (GSH) (-3.7, CI 95%-6.2 to -1.2) (6) in PCOS patients, which suggested that OS might be the pathophysiological process associated with PCOS (5). Studies have demonstrated that OS-induced pro-inflammatory states might contribute to insulin resistance and subsequent atherosclerosis (5). Another study indicated that chronic inflammatory state in patients could be considered as a potential link between PCOS and type 2 diabetes mellitus (T2DM) and cardiovascular complications (7). A summary of the baseline characteristics of the included studies is shown in **Tables 1**, **2**. Magnesium supplementation has been shown to improve insulin resistance and OS in T2DM patients (15) and subjects with metabolic syndrome (16, 17). Magnesium, as the second messenger for insulin action, regulates the auto-phosphorylation of insulin receptor to improve its sensitivity (18, 19) and reduces blood sugar levels by facilitating glucose transport. In a randomized controlled trial (RCT), patients with T2DM who took oral magnesium supplementation every day for 16 weeks were found to have a reduction in the HOMA-IR index (3.8 ± 1.1 vs. 5.0 ± 1.3, P=0.005) and the fasting glucose levels (8.0 ± 2.4 vs. 10.3 ± 2.1, P=0.01) (20). Furthermore, a cross-sectional study assessed the association between the baseline value of serum magnesium and OS in obese and non-obese women, suggesting that magnesium concentration may influence lipid peroxidation (21). Another RCT showed that magnesium co-supplementation for 6 weeks obviously reduced the levels of OS markers in pregnant women with gestational diabetes (22). Emerging evidence has suggested that serum magnesium decreased in obese patients with PCOS (18), therefore, the intake of magnesium supplements in this subset of clinical population has attracted great clinical attention. Existing studies have concluded that magnesium supplementation alone had no significant effect on the serum levels of glycemic and lipid parameters in PCOS patients (10, 11). Magnesium combined with other supplements have been reported to show inconsistent effects on the biomarkers of inflammation, OS and metabolism in PCOS patients (9, 12, 13). Moreover, there is currently no systematic review evaluating the effects of magnesium supplementation on patients with PCOS. We hypothesized that magnesium supplementation could improve the markers for OS and metabolic disorders in PCOS patients, thereby increasing ovulation rates and significantly improving the clinical outcomes for these patients. This study was based on the above assumptions to review and analyze the existing literature to investigate the impact of magnesium supplementation on the markers of OS and metabolism in PCOS patients and to provide a rationale for the clinical treatment of PCOS.

**Table 1:** Summary the characteristics of studies on the impact of magnesium supplementation alone on PCOS.

Author;	Study	Study	Diagnosis	mean age		Interventions	Dose; timing	Comparator	Sample Size		Outcome (Measure)	Key Results
Year; Country.	duration	design	criteria of PCOS	Intervention	Control				Intervention	Control		
				**Mean ± SD**	**Mean ± SD**							
Mousavi (8);	8 weeks	RCT	Rotterdam criteria	25.57 ± 4.88	26.20 ± 5.72	magnesium	a 250-mg magnesium plus placebos	placebo	21	20	hs-CRP (immunoturbidometry method);	No significant difference of treatment on serum levels of hs-CRP, MDA and TAC.
2021; Iran											MDA (thiobarbituric acid reactive substance method);	
											TAC (colorimetric method)	No significant difference on
											Insulin (ELISA method);	
			Rotterdam				a 250-mg magnesium					
Alizadeh	8 weeks	RCT		28.22 ± 6.38	26.20 ± 5.72	magnesium		placebo	21	20		
(9);2021; Iran			criteria				plus placebos				HOMA-IR (the suggested formulas); TG (colorimetric method);	study outcome measures.
											TC (colorimetric method); HDL (colorimetric method); LDL (Friede-wald formula)	
Farsinejad-Marj (10);	8 weeks	RCT	Rotterdam criteria	26.32 ± 3.92	26 ± 5.06	magnesium	250 mg magnesium and 47 mg calcium	placebo	30	30	Insulin (ELISA method);	No significant differences in serum glucose and lipid markers.
2020; Iran											HOMA-R (the suggested formula); TG (commercial available kits); TC (commercial available kits); HDL (commercial available kits); LDL (commercial available kits)	
											TG (unclear);	
												No significant changes in
Muneyyirci-			Rotterdam				400mg; twice daily					
	12 weeks	RCT		unclear	unclear	magnesium		Spironolactone/	10	14;12		
Delale (11);			criteria					Glucophage			TG (unclear);	TC, TG and HDL levels.
2013; USA											HDL (unclear)	

**Table 2:** Summary the characteristics of studies on the impact of combined magnesium supplement on metabolism of PCOS.

AuthorYear; Country.	Studyduration	Studydesign	DiagnosisCriteria of	Age		Interventions	Dose; timing	Comparator	Sample Size		Outcome (Measure)	Key Results
				Intervention	Control				Intervention	Control		
			PCOS	Mean ± SD	Mean ± SD							
Mousavi (8);2021; Iran	8 weeks	RCT	Rotterdamcriteria	25.57 ± 4.88	26.20 ± 5.72	MagnesiumplusMelatonin	250-mg/day magnesiumplus 6-mg/day melatonin	placebo	22	20	hs-CRP (immunoturbidometry method);MDA (the thiobarbituric acid reactivesubstance spectrophotometric test);TAC (colorimetric method)	Compared with the placebo, nosignificant effect of treatment onserum levels of hs-CRP, MDAand TAC(P>0.05).
ShokrpourM (12);2019; Iran	12 weeks	RCT	Rotterdamcriteria	27.2 ± 7.1	26.0 ± 3.7	Magnesiumplus vitamin E	250 mg/day magnesiumand 400 IU/day vitaminE	placebo	30	30	hs-CRP (ELISA kit (LDN, Nordhorn, Germany));MDA (thiobarbituric acid reactive substance method);TAC (ferric reduction antioxidant power method);	Compared with the placebo,significantly reduced in serum hs-CRP (3.1 ± 1.7 vs. 3.7 ± 1.5,β −0.67mg/L; 95%CI, −1.20,−0.14; P= 0.01) and increased inTAC levels (514.5 ± 77.3 vs.590.7 ± 52.2, β 66.32mmol/L;95%CI, 43.80, 88.84; P< 0.001).
AfsharEbrahimi F (13);2018; Iran	12 weeks	RCT	Rotterdamcriteria	18-40 years	18-40 years	Magnesiumplus zinc	250 mg magnesium plus220 mg of zinc, twice aday	placebo	30	30	hs-CRP (ELISA kit);MDA (the thiobarbituric acid reactivesubstance spectrophotometric test);TAC (the method of ferric-reducing antioxi-dant power)	Compared with the placebo,significantly reduced in hs-CRP(0.3 ± 0.2 vs.-1.8 ±0.2,P<0.001).No significant change in TAClevels (P=0.14).
Maktabi M (14); 2018;Iran	12 weeks	RCT	Rotterdamcriteria	23.8 ± 5.7	24.8 ± 4.8	Magnesium-Zinc-Calcium-Vitamin D	100 mg magnesium, 4mg zinc, 400 mg calciumplus 200 IU vitamin D,twice a day	placebo	30	30	hs-CRP (ELISA kit);MDA (the thiobarbituric acid reactive substancespectrophotometric test);TAC (ferric reducing antioxidant power method)	Compared with the placebo,significantly reduced in hs-CRP(0.2 ± 0.1 vs. -0.8 ± 0.1, P<0.001).No significant effect in plasmaMDA(P=0.95) and TAC concentrations(P=0.15).

## Methods

The design and reporting in this review were conducted according to the Preferred Reporting Items for Systematic Review and Meta-Analyses (PRISMA) statements (23).

### Literature search strategy

The literature was comprehensively searched by two independent reviewers from inception to January 2022 in the following electronic databases: PubMed; Cochrane Library databases; Embase; Web of science; Chinese Biological Medicine Digest (CBMD); China National Knowledge Infrastructure (CNKI); Chinese Scientific Journals Full-Text Database (VIP) Database; and Wanfang Database. We also searched ClinicalTrials.gov and Google Scholar to identify unpublished or further potential studies. The following were the search words that included both Medical Subject Headings (MeSH) terms and Free Words: “Polycystic Ovary Syndrome”; “Polycystic Ovary”; “stein-leventhal”; “sclerocystic ovary”; “PCO”, “magnesium”; “mg2”; “Randomized Controlled Trials as Topic”; “controlled clinical trial” OR “clinical trials randomized”; and “placebo”. All the potentially eligible studies were considered for review, regardless of their language and date of publication.

### Inclusion and exclusion criteria

The inclusion criteria were as follows (1): participants: PCOS diagnosed based on the Rotterdam Criteria (regardless of race or region) (2); intervention: magnesium supplementation or co- supplementation (3); outcome: markers of inflammation, OS, blood glucose, or serum lipid (4); study design: RCTs. The exclusion criteria were as follows (1): PCOS patient aged <18 years (2); the full-text of the article was unavailable to the investigators.

### Study selection

The literature was independently screened by two researchers and all the discrepancies were resolved by involving a third reviewer and through discussion or by reaching a consensus. Firstly, all the retrieved literatures were imported into Endnote X9 software for the removal of duplicate studies. Secondly, according to the inclusion and exclusion criteria, the titles and abstracts of the relevant papers were screened. Finally, the full text was read to identify the included studies and the reasons for exclusion were recorded. Prior to the formal selection, a pilot of 50 random sample citations were conducted until sufficient agreement could be reached.

### Data extraction

The relevant information was independently extracted by two reviewers, utilizing the standardized, pre-defined table, which was compiled using Microsoft Excel 2019. All discrepancies were resolved by bringing in a third examiner and a consensus was reached. The extracted data included the following information (1): basic information, including first author, study publication year, country and study design (2); characteristics of the study population such as the sample size, age and BMI (3); details of interventions and control, including the type, dose and timing (4); outcomes: C-reactive protein (CRP); malondialdehyde (MDA), total antioxidant capacity (TAC); insulin, homeostatic model assessment for insulin resistance (HOMA-IR), triglycerides (TG), total cholesterol (TC), high density lipoprotein-cholesterol (HDL-C), and low density lipoprotein-cholesterol (LDL-C). If the specific data could not be extracted from the included literature, the corresponding authors of the study were contacted.

### Assessment of the risk of bias

The risk of bias was independently assessed by two reviewers for each study using the criteria outlined in the Cochrane handbook for Systematic Reviews of Interventions and adjudication, on the advice of a third reviewer, who was brought in to address any discrepancies (24). Six specific domains associated with the risk of bias were assessed (1): random sequence generation and allocation concealment (2); blinding of the participants personnel (3); blinding of outcome assessment (4); incomplete data assessment (5); selective reporting and (6) other bias. The author’s judgments were divided into “low”, “high” or “unclear” risk of bias. Assessment of the risks of bias are shown in **Figure 2**.

### Data synthesis

As the markers of inflammation, OS, and metabolism were all continuous variables, we reported them using the mean and standard deviation (SD) of changes with 95% CIs between the baseline and the end of the study. When the changes from the baseline measurements and post-intervention values were not described, the difference between the final and the baseline means were calculated. We estimated the changes in standard deviations by using the following formula: SD change = sqrt (SD ^2^ + SD ^2^-(2* corrt × SD × SD)), where the correlation coefficient was calculated as corr = (SD ^2^ + SD ^2^- SD change^2^)/(2× SD 1 × SD 2). The interquartile range (IQR) and medians were provided, and we converted the medians into the missing mean and calculated the SD. As clinical and methodological heterogeneity was expected in the study design, characteristics of the participants, interventions and outcome measures, we used the random-effects model (25). We measured the heterogeneity using I- squared (Higgins I^2^), with a threshold value of 25%, 50%, or more than 75% being considered as low, moderate, or substantial heterogeneity, respectively. Heterogeneity in the study designs and inconsistency in the interventions prevented us from conducting a meta-analysis of the results, which could have led to a misleading summary estimate.

### Assessment of the quality of the evidence

We used the GRADE approach to assess the overall certainty of the evidence for each outcome. When deciding whether to downgrade or upgrade the certainty of the evidence for each outcome, we assessed the following factors: Downgrade: risk of bias; inconsistency; indirectness; imprecision; and publication bias. Upgrade: large effect; dose–response gradient; and plausible confounding effect. The quality of the evidence were divided into four grades: high; moderate; and low or very low based on these domains (26).

## Results

### Literature selection results

**Figure 1:**
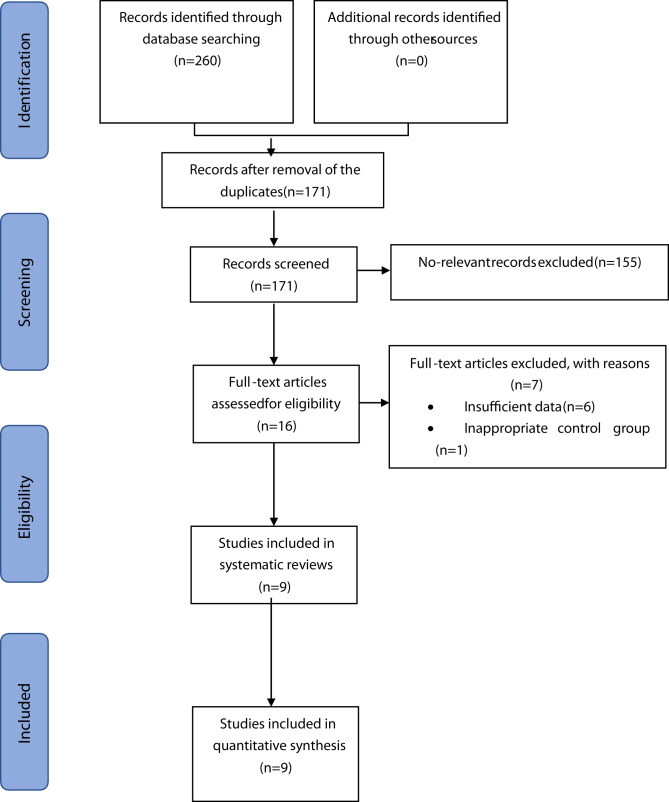
Flow chart of the study selection process.

The initial literature search found 260 records, of which 89 were excluded due to duplicate items. Among the remaining 171 articles whose abstracts were fully screened, 155 articles were further excluded due to irrelevant content for the systematic review. Sixteen studies were selected for full text reading after analysis of the abstract; six studies were excluded because they did not report any outcome measures; and one study lacked the control group. Finally, according to the inclusion and exclusion criteria, nine RCTs were included in the current systematic review (8–14, 27, 28). The detailed flowchart showing the steps for the literature retrieval process is illustrated in **Figure 1**.

### Included studies and Characteristics

A summary of the baseline characteristics of the included studies is shown in **Tables 1**, **2**. The 9 RCTs included 363 participants with PCOS. All the studies defined PCOS based on the Rotterdam criterion, eight of the studies involved subjects aged between 18-40 years, and one study lacked information regarding the baseline characteristics. Eight studies were conducted in Iran and one study was conducted in USA. Four of the nine studies had reported the impact of magnesium supplementation alone on the markers of OS or metabolism in PCOS patients (8–10). One of four studies had reported magnesium supplementation alone in comparison with metformin or spironolactone, and three studies had reported effect of magnesium supplementation in comparison with the placebo group in PCOS patients (11). Seven articles had reported the effect of magnesium co- supplementation (vitamin E (12, 28), melatonin (8, 9), zinc (13) or zinc-calcium-vitamin D (14, 27)) in comparison with the placebo group on PCOS. Of the nine RCTs, four had reported the impact of magnesium supplementation on the markers of OS and blood glucose levels, and five had reported its effect on the markers of serum lipid.

### Assessment of the risk of bias

**Figure 2:**
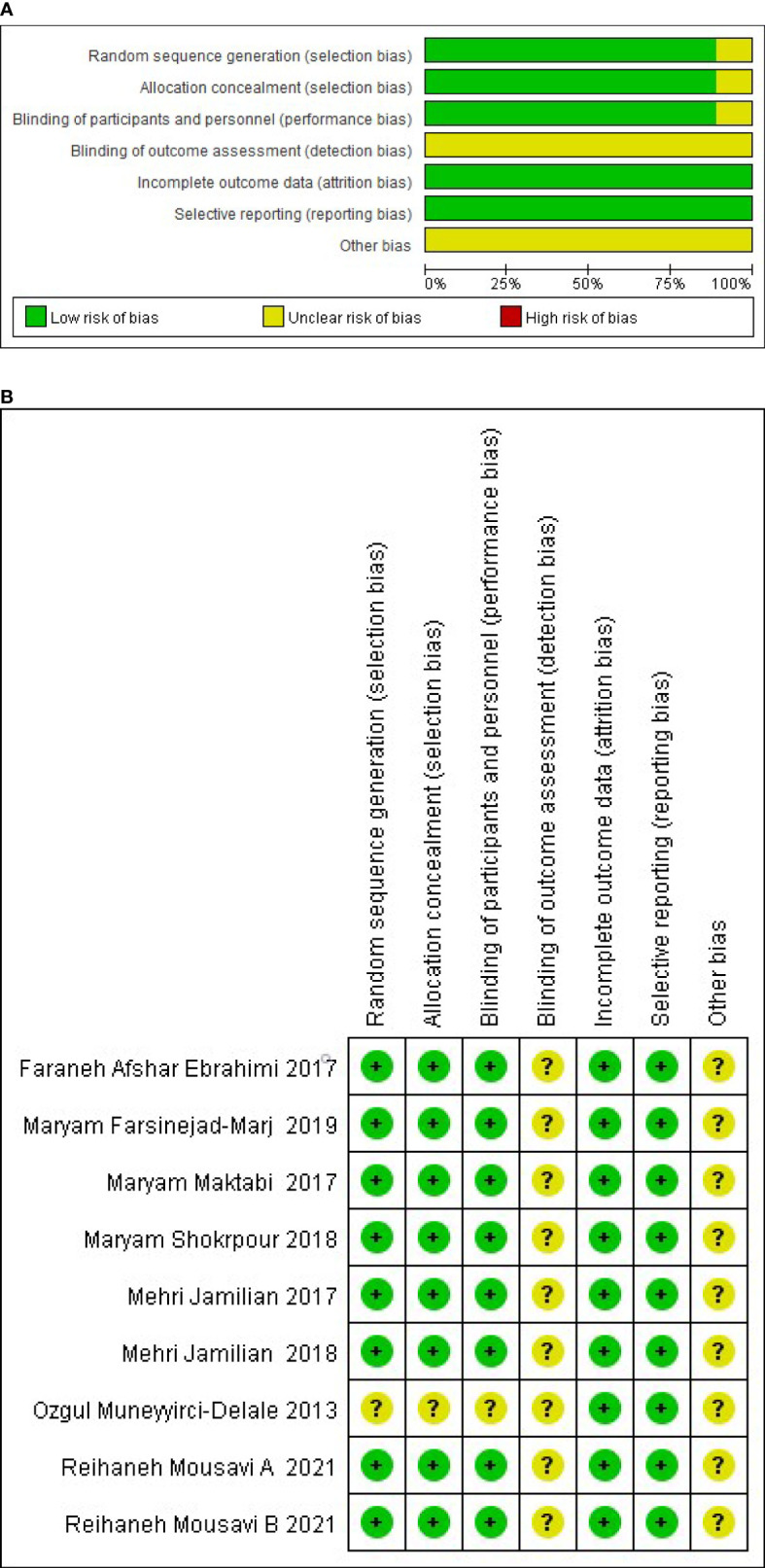
**(A)** Graph showing the risk of bias, **(B)** Summary of the risk of bias.

Among the 9 RCTs, 2 studies had reported the generation of random sequences based on random block programs developed by random allocation software (8, 9), 6 trials had performed the analysis using computer-generated random numbers (10, 12–14, 27, 28), and one study failed to provide any specific information about the generation of random sequences (11). Eight of the RCTs reported allocation concealment achieved by a trained and independent investigator (8–10, 12–14, 27, 28), and one study did not implement allocation concealment (11). None of the trials provided detailed information regarding the blinding of the outcome evaluators, but eight RCTs were double-blind (8–10, 12–14, 27, 28). The included studies reported data on the main outcomes, and also reported the specific numbers and reasons for dropped out populations. Therefore, all the studies had a low risk of attrition and reporting bias. The risk of methodological bias is presented in **Figure 2**.

### Overview of outcome measures

#### Effects of magnesium supplementation alone on the biomarkers of inflammation and OS in PCOS

##### Patients

Magnesium supplementation alone did not significantly improve the levels of inflammation or OS markers in PCOS patients. Four studies that examined the impact of magnesium supplementation alone on PCOS patients are summarized in **Table 1**. One of studies included 41 subjects who reported the effect of magnesium supplementation alone on PCOS by measuring the serum levels of hs-CRP, MDA and TAC (8). The intervention group took a 250-mg/day magnesium oxide for 8 weeks. After the 8- week intervention, magnesium supplementation was found to have no significant difference in the levels of serum hs-CRP (−0.37 (−8.98) vs. 0.07 (1.86), P=0.345), MDA (0.19 ± 0.68 vs. -0.31 ± 0.80, P=0.515) and TAC (−0.09 ± 0.22 vs. −0.13 ± 0.20, P=0.001) when compared to the placebo gr oup.

#### Effects of magnesium supplementation alone on the markers of metabolism in PCOS patients

Two RCTs (9, 10) including 101 people were assessed for the impact of oral magnesium supplementation on blood glucose and serum lipid markers after 8 week treatment. No significant differences in the levels of insulin (0.45 (7.15) vs. 0.58 (9.06), P=0.169; 13.42 ± 2.36 vs. 14.8 ± 2.23, P=0.08), HOMA-IR (0.09 (7.08) vs. 0.10 (7.63), P=0.355; 3.19 ± 0.66 vs. 3.50 ± 0.63, P=0.11), TG (1.72 ± 50.30 vs. -16.33 ± 44.88,P=0.158; 115.32 ± 17.15 vs.137.38 ± 15.7,P=0.89), TC (-4.66 ± 20.71 vs. -7.67 ± 19.54; 190.39 ± 7.88 vs. 201.35 ± 7.44, P=0.89), HDL (1.54 ± 4.72 vs. 1.19 ± 2.91,P=0.615; 45.61 ± 1.4 vs. 43.95 ± 1.32, P=0.35) and LDL (-6.42 ± 20.62 vs. -6.86 ± 18.05, P=0.07; 121.7 ± 6.78 vs. 129.93 ± 6.4, P=0.34) were found in the two studies, as compared to the placebo group. The other study included 36 subjects and did not have a placebo group, and the intervention was twice daily dose of 400 mg magnesium oxide for 12 weeks. However, there was no significant difference in the levels of TG (88.1 ± 16.4 vs. 81.9 ± 14), TC (187.1 ± 10.2vs. 182.7 ± 9.8) and HDL (47.1 ± 3.7vs. 45.7 ± 3.7) levels between the baseline values and the post-treatment values (11). The details are shown in **Table 1**.

#### Effects of magnesium co-supplementation on the biomarkers of inflammation and OS in PCOS

##### Patients

Among the seven selected literatures, four studies (8, 12–14) included 222 subjects who reported the impact of magnesium co-supplementation on the markers of OS (**Table 3**). The interventions were varied, which were a 250-mg/day magnesium combined with two 3-mg/day melatonin for 8 weeks (8); a 250mg/day magnesium combined with 400IU/day vitamin E for 12 weeks (12); 250-mg of magnesium combined with 220-mg of zinc twice a day for 12 weeks (13) and 100 mg magnesium, 4 mg zinc, 400 mg calcium alone with 200 IU of vitamin D supplements twice a day for 12 weeks (14).

**Table 3:** Summary the characteristics of studies on the impact of combined magnesium supplement on inflammation and OS of PCOS.

AuthorYear; Country.	Studyduration	Studydesign	DiagnosisCriteria of	Age		Interventions	Dose; timing	Comparator	Sample Size		Outcome (Measure)	Key Results
				Intervention	Control				Intervention	Control		
			PCOS	Mean± SD	Mean ± SD							
Alizadeh (9); 2021; Iran	8 weeks	RCT	Rotterdam	28.22 ± 6.38	26.20 ± 5.72	Magnesium	A 250-mg	placebo	22	20	Insulin (ELISA method);	Compared to the baseline values, a significant
			criteria			plus	magnesium plus				HOMA-IR (the suggested formulas);	decrease in insulin (GMD: -1.11 (mIU/mL)
						Melatonin	6-mg melatonin				TG (colorimetric method);	(percent change: -15.99)), HOMA-IR (-0.28 (-
											TC (colorimetric method);	18.66)), cholesterol (mean difference: -16.08
											HDL (colorimetric method);	(mg/dl) [95%CI -24.24, -7.92]), LDL-C
											LDL (Friede-wald formula);	(-18.96 (mg/dl) [-28.73, -9.20]) and increase in
												HDL-C levels (2.19(mg/dl) [0.61,3.77])
												(P<0.05).
Jamilian M (28); 2019; Iran	12 weeks	RCT	Rotterdam	29.2 ± 7.2	28.3 ± 3.8	Magnesium	250 mg/day	placebo	30	30	Insulin (ELISA kit);	Compared with the placebo, significantly
			criteria			plus vitamin E	magnesium and				HOMA-IR (suggested formulas);	reduced in insulin (1.6 ± 3.7 vs.-1.1± 3.0
							400 mg/day				TG (enzymatic kits);	μIU/ml, P=0.003), HOMA-IR (0.4 ± 0.9 vs. -
							vitamin E				TC (enzymatic kits);	0.2 ± 0.7, P=0.002), TG (6.7 ±22.2 vs.
											HDL (enzymatic kits);	-15.0 ± 24.4 mg/dl, P=0.001) and TC levels
											LDL (enzymatic kits);	(8.1 ± 26.6 vs. -7.0 ± 32.6 mg/dl, P=0.05).
Jamilian M (27); 2017; Iran	12 weeks	RCT	Rotterdam	18-40 years	18-40 years	Magnesium-	100 mg	placebo	30	30	Insulin (ELISA kit);	Compared to the placebo, a significant
			criteria			Zinc-Calcium-	magnesium, 4				HOMA-IR (suggested formulas);	decrease in insulin (0.4 ± 2.8 vs. -1.9 ± 4.6
						Vitamin D	mg zinc, 400 mg				TG (enzymatic kits);	μIU/ml, P=0.01), HOMA-IR(0.1 ±0.6 vs. -0.4±
							calcium plus 200				TC (enzymatic kits);	1.0 mg/dl, P=0.02), TG(8.9 ±17.9 vs. -26.5±
							IU vitamin D				HDL (enzymatic kits);	42.9 mg/dl P<0.001), TC (11.1 ±28.4 vs.
											LDL (enzymatic kits);	-4.2 ± 30.7 mg/dl P=0.04).

SD, standard deviation; CI, confidence interval; GMD, geometric means difference.

##### Inflammatory markers: hs-CRP

After the 12-week intervention, compared with the placebo group, serum hs-CRP concentrations were significantly reduced in PCOS patients after magnesium co-supplementation with vitamin E or zinc or zinc-calcium-vitamin D (3.1 ± 1.7 vs. 3.7 ± 1.5, P= 0.01; -1.8 ± 0.2 vs. 0.3 ± 0.2, P< 0.001; -0.8 ± 0.1 vs. 0.2 ± 0.1, P< 0.001) (12–14). However, no significant difference was found in serum hs-CRP levels upon intervention with the combination of magnesium and melatonin supplements PCOS patients after 8 weeks (0.34 (6.61) vs. 0.07 (1.86), P=0.345) (8).

#### Markers of OS: TAC and MDA

Compared with the placebo group, serum TAC levels were found to be increased in two studies whose interventions were magnesium co-supplementation with Vit E or melatonin (590.7 ± 52.2 vs. 514.5 ± 77.3, P<0.001; 0.09 ± 0.29 vs. -0.13 ± 0.20, P=0.001), respectively (8, 12). When controlled to the baseline values, there was no significant difference in TAC levels (51.5 ± 20.5 vs. 7.6 ± 20.5; P= 0.14; 39.6 ± 19.2 vs. -0.6 ± 19.2, P=0.15) between the two groups (13, 14). MDA levels did not change with magnesium co-supplementation (-0.1 ± 0.1 vs. 0.1 ± 0.1, P=0.18; 2.6 ± 0.2 vs. 2.5 ± 0.5, P=0.27; -0.1 ± 0.1 vs. -0.1 ± 0.1, P=0.95; 2.11 ± 0.60 vs. 1.98 ± 0.62, P=0.515) (8, 12–14).

### Effects of magnesium co-supplementation on the markers of metabolism in PCOS patients

Three (9, 27, 28) out of the seven studies included 162 people who reported the effects on blood glucose levels and lipid markers (**Table 2**). Their interventions consisted of a 250-mg/day magnesium combined with two 3-mg/day melatonin for 8 weeks (9); a 250mg/day magnesium combined with 400IU/day vitamin E for 12 weeks (28) and 100 mg magnesium, 4 mg zinc, 400 mg calcium combined with 200 IU vitamin D supplementation twice a day for 12 weeks (27).

#### Markers of blood sugar: insulin and HOMA-IR

However, the difference in the levels of insulin (-1.6 ± 0.7 vs. 0.03 ± 0.7, P=0.10) and HOMA –IR (-0.3 ± 0.1vs.0.02± 0.1, P =0.08) between the treated and placebo groups were non-significant, when they controlled the baseline values of the biochemical variables, age and baseline BMI (27). After 8 weeks of combined magnesium and melatonin supplementation, insulin (5.83 (1.50, 11.00) vs. 6.98 (1.50, 22.40), P=0.169) and HOMA-IR (1.22(0.32, 2.61) vs. 1.41 (0.32, 5.83), P=0.355) values were not significantly different in comparison with the placebo, but were significantly reduced as compared to the baseline values (5.83 (1.50, 11.00) vs. 6.94 (1.70, 21.00), P=0.01) (1.22(0.32, 2.61) vs. 1.50 (0.37, 15.00), P=0.006) (9).

#### Blood lipid markers: TG, TC, HDL and LDL

Compared with the placebo group, a combination of supplementation with magnesium and vitamin E or magnesium-zinc-calcium- vitamin D significantly reduced TG (110.0 ± 55.0 vs. 134.7 ± 68.9, P=0.001; 95.1 ± 38.6 vs. 126.6 ± 55.5, P<0.001) and TC (174.5 ± 32.2 vs. 193.2 ± 33.7, P=0.05; 155.9 ± 28.2 vs. 174.4 ± 34.2, P=0.04) levels, but there were no significant differences in HDL (51.1 ± 8.6 vs. 52.0 ± 10.9, P=0.92; 48.5 ± 7.1 vs. 48.4 ± 9.3,P=0.77) and LDL (101.4 ± 30.4 vs.114.2 ± 38.9, P=0.15; 88.4 ± 24.4 vs. 100.7 ± 31.1;P=0.21) levels (27, 28). As compared to the baseline values, after 8 weeks of magnesium-melatonin supplementation, TC (159.73 ± 23.54 vs. 175.81 ± 34.67, P=0.001) and LDL (82.33 ± 15.72 vs. 101.30 ± 28.55, P=0.001) levels were s ignificantly reduced.

### Quality of the evidence

GRADE evidence summary indicated that the quality of the ten outcome measures was “moderate” or “low”. Reasons for the loss of quality of the evidence included inconsistencies (differences in the interventions and timing) in the included studies and the greater risk of bias. The results for the assessment of the quality of the evidence are displayed in **Table 4**.

**Table 4:** Quality of evidence in the included systematic reviews with GRADE.

Outcomes	No. of studies	Risk of bias	Inconsistency	Indirectness	Imprecision	Publication bias	OverallOutcomesCertainty evidence	No. of Participants [invention]	No. of Participants [control]
**hs-CRP**	(5 RCTs)	not serious ^a^	Serious ^b, c^	not serious	not serious	not serious	Moderate ⨁⨁⨁◯	133	130
**TAC**	(5 RCTs)	not serious ^a^	Serious ^b, c^	not serious	not serious	not serious	Moderate ⨁⨁⨁◯	133	130
**MDA**	(5 RCTs)	not serious ^a^	Serious ^b, c^	not serious	not serious	not serious	Moderate ⨁⨁⨁◯	133	130
**insulin**	(5 RCTs)	not serious ^a^	Serious ^b, c^	not serious	not serious	not serious	Low ⨁⨁◯◯	133	130
**HOMA- IR**	(5 RCTs)	not serious ^a^	Serious ^b, c^	not serious	not serious	not serious	Moderate ⨁⨁⨁◯	130	133
**TG**	(6 RCTs)	Serious ^d^	Serious ^b, c^	not serious	not serious	not serious	Low⨁⨁◯◯	143	144
**TC**	(6 RCTs)	Serious ^d^	Serious ^b, c^	not serious	not serious	not serious	Low ⨁⨁◯◯	143	144
**HDL**	(6 RCTs)	Serious ^d^	Serious ^b, c^	not serious	not serious	not serious	Low ⨁⨁◯◯	143	144
**LDL**	(5 RCTs)	not serious ^a^	Serious ^b, c^	not serious	not serious	not serious	Moderate ⨁⨁⨁◯	133	130

^a^The risk of bias in the original studies were low. ^b^Participants from Iran contributed nearly 90% of the data in the systematic review. Therefore, the results apply primarily to the Iranian population. ^c^Interventions and timings were inconsistent across the different included studies. ^d^One of the studies had a higher risk of bias, while the others had a lower risk.

## Discussion

We conducted a systematic review of the RCTs to investigate the extent to which magnesium supplementation affected the markers of inflammation, OS, blood glucose and serum lipid in PCOS patients. When magnesium was co-supplemented with other agents, magnesium was found to improve the inflammatory response, insulin resistance and lipid metabolism. However, we could not find any beneficial effects of magnesium supplementation alone on the markers of OS and metabolism in PCOS patients. Serum concentrations of magnesium influences glucose homeostasis, lipid synthesis, ATP metabolism and so on. Recent evidence indicates that the presence of low-grade inflammation in PCOS patients increases the circulating levels of inflammatory mediators, such as TNF-α and CRP, which are one of the potential links between PCOS and long-term metabolic disorders or cardiovascular complications (7). A meta-analysis involving 18 RCTs showed that magnesium supplements had no impact on serum CRP levels, and based on dose-response assessments reported that the relationship between magnesium supplementation dose or timing and serum CRP concentration was non-linear (29). The above results were consistent with our findings, which suggested that magnesium supplementation alone did not improve serum CRP levels. Magnesium supplementation in children with allergic asthma showed that magnesium exerted antioxidant activity and affected the glutathione reducing system (30). However, magnesium supplementation alone did not improve markers of OS in PCOS patients (8). Magnesium acts as a cofactor, regulating the activity of rate-limiting enzymes involved in glycolysis, glucose homeostasis, and insulin signaling (31). In addition, magnesium deficiency is involved in the pathogenesis of dyslipidemia by increasing the activity of lecithin cholesterol acyltransferase and decreasing the activity of lipoprotein lipase (32). Patients with T2DM who took 250 mg magnesium for 3 months, showed significant improvement in their insulin levels (15.56 to 12.18, P < 0.001) and HOMA-IR (6.16 to 4.44, P <0.001) as compared to the baseline values (33). In another RCT, after 16 weeks of magnesium supplementation, the fasting glucose and triglyceride levels were found to be decreased in patients with metabolic syndrome (34). However, after 12 weeks of magnesium oxide supplementation, HOMA-IR increased (1.9 ± 4.0 vs. 0.2 ± 2.5, P = 0.04) and the lipid profile (TG/TC/HDL/LDL) did not improve significantly in hypo-magnesemic patients diagnosed with T2DM and patients with early-stage nephropathy (35). Several factors may explain the results that we obtained. In the magnesium group, the enrolledpopulation had sufficient baseline magnesium status, hence, the beneficial effects of magnesium supplementation on inflammation was difficult to be observed in this group (8). As a matter of fact, oral magnesium supplementation may be more effective in individuals with magnesium-deficiency (16). Another important reason is that the proximal small intestine is the primary site for magnesium digestion and absorption, therefore, gastrointestinal disorders may have a potential impact on the beneficial effects of oral magnesium supplements (29). In the literature included in this systematic review, compared with the placebo group, serum hs- CRP levels, insulin and HOMA-IR did not decrease in the magnesium group after magnesium and melatonin supplementation (8). Melatonin, as a regulator between inflammatory cells, has a powerful antioxidant potential with the ability to reduce the oxidative environment associated with chronic inflammation (36). In addition, melatonin reduces OS owing to its ability to scavenge free radicals, which is directly related to the concentration of melatonin (37). One trial showed that melatoninadministration significantly reduced the serum levels of hs-CRP (-0.61 mg/L; P=0.001) and MDA (- 0.25 µmol/L; P< 0.001), and significantly increased the serum levels of TAC (106.07 mmol/L; P< 0.001) (38). Another meta-analysis of six RCTs reported that melatonin supplementation significantly reduced the levels of CRP in patients with metabolic syndrome (39). We concluded that the combination of magnesium and melatonin did not reduce serum CRP levels, but increased TAC levels, which could be potentially be dependent on the dose and timing of melatonin supplementation (8). In the literature included in this systematic review, as compared with the baseline values, there was a reduction in the serum levels of insulin, HOMA-IR, cholesterol and LDL-C and increase in HDL-C levels after 8 weeks of magnesium and melatonin supplementation (9). Melatonin has been reported to have beneficial effects on hyperglycemia, insulin resistance and dyslipidemia. Daily intake of 10mg melatonin for 12 weeks by diabetic patients undergoing hemodialysis showed beneficial effects on blood glycemic control, levels of inflammatory markers and OS (40). Melatonin also plays a key role in regulating lipid metabolism, possibly by increasing the activity of lipoprotein lipase and LDL receptor and decreasing lipolysis to improve dyslipidemia (41, 42). Melatonin may be classified as a vitamin which is more effective in combination with magnesium than either of them alone (43). Although there are only few studies comparing the effects of magnesium and/or melatonin supplementation on the levels of the markers of metabolism and inflammation in PCOS patients, combined magnesium and melatonin supplementation was found to be more effective in improving the markers of OS, lipid profile, and insulin resistance in PCOS patents (8, 9).. Vitamin E is an antioxidant that boosts the free radical defense system and is good for improving glucose transport and insulin sensitivity (44). Vitamin E treatment has also been reported to decrease plasma LDL and TG levels, inhibit lipid peroxidation and reduce blood viscosity. In patients with T2DM, vitamin E supplementation for three months was shown to reduce the levels of serum glucose (8.3 ± 0.3 vs. 7.5 ± 0.2, P> 0.05), TG (2.27 ± 0.08 vs. 1.67 ± 0.09, P< 0.02), TC (6.74 ± 0.09 vs. 5.50 ± 32 0.10, P< 0.05) and LDL-C (4.73 ± 0.11 vs. 3.67 ± 0.07, P< 0.04) (45). In another RCT, serum magnesium levels in patients with essential hypertension were also found to be significantly increased after 4 weeks of vitamin E supplementation (1.71 ± 0.04 to 1.99 ± 0.05 mmol/L; P,0.01) (46). Additionally, one study analyzed the MDA and lipid metabolism parameters in diabetic rats treated with a combination of magnesium and vitamin E or magnesium alone. After 8 weeks, the two groups showed significant reduction in the levels of MDA, plasma TC and LDL, however, the effects of magnesium combined with VE were superior than magnesium supplementation alone. These findings suggested that magnesium-vitamin E co-supplementation had a better impact on OS and lipid metabolism in diabetic rats. Zinc also plays a vital role in maintaining the redox homeostasis, and has been reported to exert antioxidant and protective effects on reactive oxygen species (47). Zinc is widely involved in insulin secretion and blood pressure regulation. The imbalance in zinc homeostasis is closely associated with the development of cardiovascular disorders and production of reactive oxygen species (48). Apreviously published study showed that after 8 weeks of zinc supplementation, plasma MDA levels were significantly decreased in Patients with Hypothyroidism. (−0.09 ± 1.31 vs. + 2.34 ± 5.5, P= 0.04) (49). In addition, serum hs-CRP levels were significantly decreased and plasma TAC levels were increased after 12 weeks of magnesium and zinc co-supplementation (13). However, in another RCT, the MDA and TAC levels were not found to be significantly altered after 10 weeks of magnesium, zinc, and vitamin A co-supplementation (50). Combined supplementation of magnesium and zinc had a strong synergistic effect on improving OS and inflammatory response in PCOS patients (8, 13, 49). However, since these studies were conducted in different populations, the synergistic effect of magnesium and zinc was hypothetical. Therefore, further comparison of the effects of magnesium and zinc supplementation alone and in combination needs to be tested in PCOS patients from a uniform population. Vitamin D has been shown to mediate the formation of glutathione(GSH), and the accumulation of GSH scavenges the reactive oxygen species, and the reduction in OS in turn inhibits the secretion of monocyte pro-inflammatory cytokines (51). Previous meta-analysis showed that vitamin D supplementation significantly reduced the levels of hs-CRP (SMD-1.03; p< 0.001) and MDA (SMD- 1.64; P<0.001), and increased TAC levels (SMD 0.86; P =0.03) in PCOS patients (52). Of note, the levels of hs-CRP and MDA were reduced after vitamin D co-supplementation with other nutrients, as compared with vitamin D alone (52). The primary function of vitamin D is to maintain calcium and phosphorus homeostasis and promote bone mineralization (53). Therefore, in clinical practice, calcium and vitamin D are usually administered together. Calcium and vitamin D supplementation significantly increased GSH levels (51.14 ± 131.64 vs −47.27 ± 203.63μmol/L, P =0.03) in women with gestational diabetes mellitus (54). However, in patients with sporadic colorectal adenoma, elemental calcium supplementation had no effects on circulating biomarkers of inflammation and OS (55). Compared with the placebo group, 12 weeks of supplementation with Magnesium-Zinc-Calcium-Vitamin D significantly improved OS, inflammatory factors (22), blood glucose, and serum lipid levels (TG/TC) (27) in women with gestational diabetes. The above results were consistent with those in PCOS patients. We speculated that after magnesium, zinc, calcium, or vitamin supplementation, the major reason for inconsistent outcomes could be dependent on whether the patient’s baseline values were reduced or not. If the serum levels of magnesium, zinc and calcium were reduced in the participants before any intervention, the improvement in the levels of biomarkers of OS and metabolism may have been more pronounced after supplementation.

### Limitations of the literature

Although multiple databases were thoroughly searched and there was no restriction on the language or the country, only one of the nine studies was performed in the United States, and all the others were conducted in Iran. Thus, the nature of the study population could potentially limit the generality of the findings to other ethnic groups. Based on the current study, we cannot confirm the effect of magnesium supplements on PCOS patients of other races. In addition, interventions used in the included studies for combined magnesium supplementation were inconsistent. Therefore, it is difficult to determine which supplementation was responsible for the effects and whether the combination of different supplements were synergistic. Finally, the methods used to measure the outcomes were also different, which could have led to the variations in the results. In future studies, if possible, the effect of magnesium supplementation alone and in combination with other supplements on PCOS should be directly compared, and the outcome indicators should also be measured using a uniform method, In order to determine with certainty, the beneficiary effects of specific supplements or a combination of supplements on PCOS patients and to improve the therapeutic options for PCOS patients.

## Conclusions

In conclusion, magnesium supplementation alone did not lead to a significant improvement in the markers of OS, blood glucose, or plasma lipid in PCOS patients. The serum levels of HS-CRP, insulin and HOMA-IR were significantly improved by magnesium co-supplementation with other supplements. The improvement in the lipid profile after magnesium supplementation remained controversial. Although magnesium supplementation did not significantly improve the markers of OS and metabolic disorders in PCOS patients in our included studies, the beneficial effects of magnesium were confirmed in patients with diabetes and metabolic syndrome. Magnesium in combination with vitamin E, zinc, or calcium significantly improved insulin resistance and inflammation. However, large numbers of further long-term studies are needed to confirm the association and synergy between magnesium supplements and other supplements, and to demonstrate the benefits of magnesium supplements in PCOS patients.

## Author contributions

YY developed the topic and served as the guarantor of this study. BM designed the search strategies. RL and ZL conducted literature search and draft writing. KH was responsible for topic refinement. RL, YH and KH revised the first draft multiple times and reached consensus on the final draft. All authors contributed to the article and approved the publication of the submitted version.

## Funding

This work was supported by the Regional Scientists Fund of the National Natural Science Foundation of China (grant number 81960275); Project of Lanzhou Science and Technology Bureau(2021-RC-133) and the Foundation Project of Gansu Provincial Science and Technology Department key research and development (grant number21YF5FA119).

## Conflict of interest

The authors declare that the research was conducted in the absence of any commercial or financial relationships that could be construed as a potential conflict of interest.

## Publisher’s note

All claims expressed in this article are solely those of the authors and do not necessarily represent those of their affiliated organizations, or those of the publisher, the editors and the reviewers. Any product that may be evaluated in this article, or claim that may be made by its manufacturer, is not guaranteed or endorsed by the publisher.
